# Reproducibility of a Semi-quantitative Food Frequency Questionnaire in Japanese Female Dietitians

**DOI:** 10.2188/jea.12.45

**Published:** 2007-11-30

**Authors:** Nahomi Imaeda, Nakako Fujiwara, Yuko Tokudome, Masato Ikeda, Kiyonori Kuriki, Teruo Nagaya, Juichi Sato, Chiho Goto, Shinzo Maki, Shinkan Tokudome

**Affiliations:** 1 Nagoya City School of Nutrition, Mizuho-ku, Nagoya.; 2 Nagoya City University School of Nursing, Mizuho-ku, Nagoya.; 3 Nagoya-Bunri College, Nishi-ku, Nagoya.; 4 University of Occupational and Environmental Health, Yahatanishi-ku, Kitakyushu.; 5 Department of Public Health, Nagoya City University Medical School, Mizuho-ku, Nagoya.; 6 Nagoya University Hospital, Showa-ku, Nagoya.; 7 The Aichi Prefectural Dietetic Association, Kita-ku, Nagoya, Japan.

**Keywords:** intake of foods and nutrients, Japanese female dietitians, reproducibility, semi-quantitative food frequency questionnaire

## Abstract

Objective: To examine reproducibility of assessed intake of foods and nutrients according to a semi-quantitative food frequency questionnaire (SQFFQ) in Japanese female dietitians. Subjects and Methods: An SQFFQ was self-administered to 106 (21 male and 85 female) Japanese dietitians in Aichi prefecture in autumn 1996 and the same questionnaire was repeated in autumn 1997. Reproducibility was evaluated in terms of consumption of 15 foods and energy and 30 macro- and micro-nutrients based on the SQFFQ from 84 Japanese female dietitians. Results: For intake of foods, Pearson’s correlation coefficients (CCs) with log-transformation and energy adjustment (minimum - median - maximum) ranged from 0.35 (beverages) - 0.61 - 0.71 (dairy products). ANOVA intraclass correlation coefficients (ICCs) with log-transformation and energy adjustment ranged from 0.49 (beverages) - 0.74 - 0.82 (dairy products). Spearman’s rank CCs with energy adjustment ranged from 0.43 (confectionery) - 0.57 - 0.76 (dairy products). Weighted *kappa* statistics with energy adjustment ranged from 0.34 (confectionery) - 0.49 - 0.71 (dairy products). For consumption of nutrients, Pearson’s CCs with log-transformation and energy adjustment ranged from 0.23 (zinc) - 0.55 - 0.74 (insoluble dietary fiber). ANOVA ICCs with log-transformation and energy adjustment ranged from 0.37 (zinc) - 0.70 - 0.84 (insoluble dietary fiber). Spearman’s rank CCs with energy adjustment ranged from 0.25 (zinc) - 0.56 - 0.74 (magnesium). Weighted *kappa* statistics with energy adjustment ranged from 0.25 (zinc) - 0.50 - 0.68 (insoluble dietary fiber). Conclusions: Substantially high reproducibility of consumption of foods and nutrients was attained from an SQFFQ self-administered to Japanese female dietitians.

## INTRODUCTION

In developed countries, the major causes of death are chronic/lifestyle-related diseases including cancer, cerebrovascular disease and heart disease^1^^,^^2^^)^. In Japan two-thirds of all deaths consist of these causes, which are associated with accumulation of gene aberrations caused by interaction of genetic factors and daily lifestyle. The pathogenesis proceeds in a multi-factor, multi-hit and multi-stage fashion with a long latency period. Genetic factors are of interest in terms of etiology but lifestyle is more important in that we can modify it with a view to disease prevention and health promotion.

Lifestyle factors include food intake, alcohol drinking, smoking, physical exercise, and stress. Among these, smoking is the most potent single factor contributing to chronic diseases. Food consumption also appears to play a crucial role^3^^)^, but in this case both deleterious and beneficial aspects are evident. Observations may remain inconsistent, however, because information on diet is not necessarily valid or reproducible due to fluctuations in dietary intake itself, and in procedures to obtain information on diet and study subjects.

We have developed a data-based SQFFQ to secure long-term dietary intake^4^^)^ and conducted a relative validation/calibration of consumption of nutrients as well as foods based on the SQFFQ versus findings with four season consecutive 7 day weighed diet records (abbreviated WDRs hereafter)^5^^)^. Here, we studied reproducibility/repeatability of intake of foods and nutrients by comparing results of two SQFFQs self-administered at a one-year interval to Japanese female dietitians.

## SUBJECTS AND METHODS

### Subjects and SQFFQ

We earlier designed an evidence-based SQFFQ according to multiple regression and contribution analyses, and validated the SQFFQ versus 28 day WDRs as described elsewhere^4^^-^^6^^)^.

**Figure 1.**
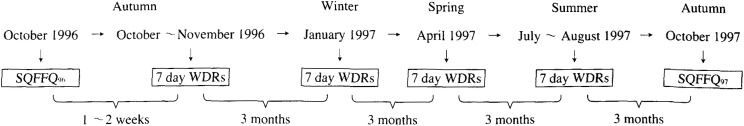
Schedule for validation/reproducibility studies of an SQFFQ.

Briefly, in autumn 1996, we recruited 106 (21 male and 85 female) middle-aged Japanese dietitians living in Aichi prefecture, Central Japan, and first mail-administered the SQFFQ (SQFFQ_96_) and surveyed consecutive 7 day WDRs approximately one week later, and then at about 3 month intervals in winter, spring and summer 1997 (Figure 1). In autumn 1997, the same questionnaire (SQFFQ_97_) was applied again.

All female dietitians completed both SQFFQ_96_ and SQFFQ_97_; however, one subject was excluded because her response on energy lay beyond 4 and 5 standard deviations from the group means with the SQFFQ_96_ and SQFFQ_97_, respectively. Since the number of male dietitians was rather small, they were not included in the present study.

Accordingly, the results are shown for 84 female dietitians with a mean age (years of age) ± standard deviation (minimum - maximum) of 47 ± 8 (32 - 66). The values for height (cm), weight (kg) and body mass index (BMI) (kg/m^2^) were 156.1 ± 5.0 (146.1 - 172.1), 52.3 ± 5.4 (38.4 - 67.8) and 21.5 ± 2.1 (16 - 29), respectively.

### Foods and Nutrients Selected

We chose fifteen foods/food groups and beverages including rice, bread, noodles and potatoes, confectionery, oil, soybean and soybean products, fish and other seafoods, meat, eggs, dairy products, green-yellow vegetables, other vegetables, seaweed, fruit, beverages, and alcohol.

In addition, energy and thirty macro- and micro-nutrients were selected, including protein, fat, carbohydrate, total dietary fiber (TDF) (soluble DF and insoluble DF), minerals (potassium, calcium, magnesium, phosphorous, iron, zinc and copper) and vitamins (carotenes and vitamins A, D, E and C).

Fat was divided into saturated fatty acids (abbreviated SFAs hereafter) ( myristic acid (14:0), palmitic acid (16:0), and stearic acid (18:0)), mono-unsaturated fatty acids (abbreviated MUFAs hereafter) (including oleic acid), poly-unsaturated fatty acids (abbreviated PUFAs hereafter), n-6 PUFAs, n-3 PUFAs and cholesterol, n-6 PUFAs were separated into linoleic acid (18: 2n-6) and arachidonic acid (20: 4n-6), and n-3 PUFAs into *α*-linolenic acid (18: 3n-3), eicosapentaenoic acid (EPA, 20: 5n-3) and docosahexaenoic acid (DHA, 22: 6n-3).

### Intake of Foods and Nutrients

For regular foods and recipes, except for type and frequency of breakfast and consumption of beverages, food frequency was categorized into eight: that is, never or seldom, 1-3 times/month, 1-2 times/week, 3-4 times/week, 5-6 times/week, once/day, twice/day, and more than twice/day. Intake of beverages was assessed in an open-ended manner. Serving sizes were half, one, 1.5 and two times the standard portion size, and an open-ended bracket was also prepared.

We ascertained average daily consumption of foods and nutrients by multiplying the food intake (in grams) or serving size and the nutrient content per 100 grams of food as listed in the Standard Tables of Food Composition, Version 4, the Follow-up of Standard Tables of Food Composition^7^^,^^8^^)^, Composition Table of Processed Foodstuff^9^^)^ and Table of Trace Element Contents in Japanese Foodstuffs^10^^)^ or per 100 grams of model recipe.

### Statistical Analysis

Firstly, we compared daily consumption of 15 foods and beverages, and energy and 30 macro- and micro-nutrients according to the SQFFQ_96_ and SQFFQ_97_. The differences of means were examined by paired t-test.

Secondly, we calculated Pearson’s correlation coefficients (abbreviated CCs hereafter), log-transformed CCs, energy-adjusted and log-transformed CCs with 95% confidence intervals^11^^-^^16^^)^, ANOVA intraclass CCs (ICCs hereafter), log-transformed ICCs, and energy-adjusted and log-transformed ICCs with 95% confidence intervals, and Spearman’s rank CCs and energy-adjusted Spearman’s rank CCs with 95% confidence intervals for intake of selected foods and nutrients with the two SQFFQs.

Thirdly, after categorizing daily consumption of the selected foods and nutrients measured in the two SQFFQs into three groups, we computed percentages of exact agreement and complete disagreement, and *kappa* and weighted *kappa* statistics with 95% confidence intervals^17^^,^^18^^)^.

## RESULTS

### Intake of Foods

Table 1 shows comparisons between daily intake of foods according to the SQFFQ_96_ and SQFFQ_97_. The values with SQFFQ_96_ were equal to or larger than those of SQFFQ_97_, except in the alcohol case. There were no statistical differences in daily consumption of foods between the two questionnaires, except for confectionery.

**Table 1.** Comparison of daily intake of selected foods according to SQFFQ_96_ and SQFFQ_97_.

Food	SQFFQ_96_		SQFFQ_97_		% difference from SQFFQ_96_
	
	Mean	SD	Mean	SD	
Rice	293 (g)	110	293	111	0%
Bread, noodles and potatoes	161	70	162	73	1%
Confectionery	39	19	34	24	-13% *
Oil	17	11	16	10	-4%
Soybean and soybean products	66	31	61	29	-8%
Fish and other seafoods	85	37	83	41	-2%
Meat	65	33	65	34	0%
Eggs	32	23	30	17	-7%
Dairy products	250	191	239	145	-5%
Green-yellow vegetables	146	83	143	79	-2%
Other vegetables	177	128	171	98	-3%
Seaweed	12	7	12	8	-1%
Fruit	164	103	157	99	-4%
Beverages	732	638	741	583	1%
Alcohol	10	25	12	31	25%

Median					-2%

* p<0.05 by paired *t*-test.

Pearson’s CCs with log-transformation and energy adjustment (minimum - median - maximum) ranged from 0.35 (beverages) - 0.61 - 0.71 (dairy products) (Table 2). ANOVA ICCs with log-transformation and energy adjustment ranged from 0.49 (beverages) - 0.74 - 0.82 (dairy products). Spearman’s rank CCs with energy adjustment ranged from 0.43 (confectionery) - 0.57 - 0.76 (daily products). Consistent amelioration was not exerted either on Pearson’s CCs or on ANOVA ICCs by log-transformation with/without energy-adjustment. Spearman’s rank CCs were not improved by energy-adjustment, either.

**Table 2.** Pearson’s CCs, ANOVA ICCs and Spearman’s rank CCs between daily intake of selected foods based on SQFFQ_96_ and SQFFQ_97_.

	Pearson’s CCs				ANOVA ICCs				Spearman’s rank CCs		
			
Food		Log- transformed	Log- transformed, energy- adjusted	95% CIs		Log- transformed	Log- transformed, energy- adjusted	95% CIs		Energy- adjusted	95% CIs
Rice	0.73	0.70	0.67	(0.54 - 0.78)	0.84	0.82	0.80	(0.69 - 0.87)	0.70	0.69	(0.56 - 0.79)
Bread, noodles and potatoes	0.51	0.60	0.49	(0.31 - 0.64)	0.68	0.75	0.65	(0.46 - 0.77)	0.52	0.50	(0.32 - 0.64)
Confectionery	0.61	0.65	0.39	(0.19 - 0.56)	0.75	0.77	0.58	(0.36 - 0.73)	0.65	0.43	(0.23 - 0.59)
Oil	0.72	0.63	0.48	(0.30 - 0.63)	0.84	0.77	0.64	(0.45 - 0.77)	0.67	0.50	(0.32 - 0.65)
Soybean and soybean products	0.57	0.52	0.54	(0.37 - 0.68)	0.73	0.68	0.70	(0.54 - 0.81)	0.59	0.57	(0.40 - 0.70)
Fish and other seafoods	0.66	0.74	0.62	(0.46 - 0.73)	0.79	0.84	0.76	(0.63 - 0.85)	0.69	0.64	(0.50 - 0.75)
Meat	0.59	0.68	0.67	(0.53 - 0.77)	0.74	0.81	0.80	(0.69 - 0.87)	0.61	0.58	(0.41 - 0.70)
Eggs	0.75	0.65	0.56	(0.39 - 0.69)	0.83	0.79	0.72	(0.57 - 0.82)	0.73	0.54	(0.36 - 0.67)
Dairy products	0.64	0.73	0.71	(0.59 - 0.80)	0.76	0.84	0.82	(0.73 - 0.89)	0.71	0.76	(0.65 - 0.84)
Green-yellow vegetables	0.66	0.65	0.61	(0.45 - 0.73)	0.80	0.79	0.76	(0.63 - 0.84)	0.56	0.57	(0.41 - 0.70)
Other vegetables	0.62	0.56	0.55	(0.38 - 0.68)	0.75	0.72	0.69	(0.53 - 0.80)	0.53	0.49	(0.31 - 0.64)
Seaweed	0.53	0.60	0.65	(0.50 - 0.76)	0.69	0.75	0.78	(0.66 - 0.86)	0.57	0.60	(0.44 - 0.72)
Fruit	0.50	0.66	0.61	(0.46 - 0.73)	0.67	0.78	0.74	(0.60 - 0.83)	0.62	0.60	(0.45 - 0.72)
Beverages	0.72	0.35	0.35	(0.15 - 0.53)	0.84	0.50	0.49	(0.22 - 0.67)	0.50	0.48	(0.30 - 0.63)
Alcohol	0.38	0.60	0.60	(0.44 - 0.72)	0.54	0.75	0.76	(0.64 - 0.85)	0.62	0.71	(0.58 - 0.80)

Median	0.62	0.65	0.61		0.75	0.78	0.74		0.62	0.57	

Agreement with energy adjustment ranged from 44% (confectionery and other vegetables) - 55% - 73% (dairy products) and disagreement ranged from 4% (rice and dairy products) - 8% - 11% (confectionery) (Table 3). *Kappa* statistics with energy adjustment ranged from 0.16 (confectionery and other vegetables) - 0.33 - 0.59 (dairy products). Weighted *kappa* statistics ranged from 0.34 (confectionery) - 0.49 - 0.71 (dairy products). The values of weighted *kappa* statistics were consistently better with *kappa* statistics.

**Table 3.** Agreement, disagreement and *kappa* statistics according to tertile classification of daily intake of selected foods based on SQFFQ_96_ and SQFFQ_97_.

Food	Agreement (%)	Disagreement (%)	*Kappa*	Weighted *kappa*	95% CIs	Energy-adjusted				

						Agreement (%)	Disagreement (%)	*Kappa*	Weighted *kappa*	95% CIs
Rice	71	6	0.57	0.65	(0.49 - 0.81)	68	4	0.52	0.68	(0.55 - 0.81)
Bread, noodles and potatoes	56	8	0.34	0.48	(0.31 - 0.66)	49	8	0.23	0.43	(0.25 - 0.60)
Confectionery	57	7	0.36	0.52	(0.35 - 0.69)	44	11	0.16	0.34	(0.15 - 0.53)
Oil	56	4	0.34	0.59	(0.46 - 0.72)	55	10	0.32	0.45	(0.26 - 0.63)
Soybean and soybean products	55	5	0.32	0.55	(0.41 - 0.70)	63	8	0.45	0.54	(0.36 - 0.71)
Fish and other seafoods	61	6	0.41	0.57	(0.41 - 0.73)	60	7	0.39	0.54	(0.37 - 0.70)
Meat	58	8	0.38	0.50	(0.32 - 0.68)	55	7	0.32	0.50	(0.33 - 0.67)
Eggs	67	2	0.49	0.66	(0.53 - 0.79)	51	8	0.27	0.45	(0.27 - 0.62)
Dairy products	74	5	0.61	0.70	(0.55 - 0.84)	73	4	0.59	0.71	(0.58 - 0.85)
Green-yellow vegetables	55	10	0.32	0.45	(0.26 - 0.63)	45	7	0.18	0.43	(0.27 - 0.59)
Other vegetables	54	8	0.30	0.46	(0.29 - 0.64)	44	8	0.16	0.39	(0.22 - 0.57)
Seaweed	55	10	0.32	0.45	(0.26 - 0.63)	60	7	0.39	0.54	(0.37 - 0.70)
Fruit	51	2	0.27	0.58	(0.46 - 0.69)	58	6	0.38	0.55	(0.40 - 0.71)
Beverages	57	8	0.36	0.49	(0.31 - 0.67)	56	8	0.34	0.48	(0.31 - 0.66)

Median	57	7	0.35	0.54		55	8	0.33	0.49	

Alchol was not included because the proportion of regular drinkers was less than 25%.

### Intake of Nutrients

Table 4 lists comparisons between daily intake of energy and macro- and micro-nutrients based on SQFFQ_96_ and SQFFQ_97_. The values of SQFFQ_96_ were almost equal to or larger than those of SQFFQ_97_. There were no statistical differences in daily consumption of nutrients between the two, except for vegetable fat, PUFAs, n-6 PUFAs, linoleic acid, n-3 PUFAs, *α*-linolenic acid, n-6 PUFAs/n-3 PUFAs, vitamin E and magnesium.

**Table 4.** Comparison of daily intake of selected nutrients according to SQFFQ_96_ and SQFFQ_97_.

Nutrient	SQFFQ_96_		SQFFQ_97_		% difference from SQFFQ_96_
	
	Mean	SD	Mean	SD	
Energy (kcal)	1,756	361	1,702	415	-3%
Total protein (g)	74.7	18.2	72.7	21.0	-3%
Animal origin (g)	43.2	13.9	42.3	15.4	-2%
Vegetable origin (g)	31.6	6.7	30.4	7.2	-4%
Total fat (g)	57.2	18.1	54.8	18.3	-4%
Animal origin (g)	24.9	10.9	24.2	9.6	-3%
Fish origin (g)	6.2	2.9	6.2	3.1	-1%
Vegetable origin (g)	26.0	8.7	24.5	9.4	-6%*
Carbohydrate (g)	228.4	45.4	221.6	51.2	-3%

SFAs (mg)	16,444	6,320	15,782	5,465	-4%
MUFAs (mg)	18,880	6,391	18,505	6,532	-2%
18:1 (mg)	16,479	5,687	16,147	5,835	-2%
PUFAs (mg)	13,537	3,991	12,621	4,340	-7%**
n-6 PUFAs (mg)	10,668	3,177	10,070	3,634	-6%*
18:2n-6 (mg)	10,471	3,138	9,878	3,593	-6%*
20:4n-6 (mg)	137	51	132	50	-4%
n-3 PUFAs (mg)	2,856	933	2,543	910	-11%**
18:3n-3 (mg)	1,682	596	1,377	571	-18%**
20:5n-3 (mg)	338	162	337	175	0%
22:6n-3 (mg)	605	262	599	282	-1%
n-6 PUFAs/n-3 PUFAs	3.8	0.7	4.1	0.9	7%**
Cholesterol (mg)	341	136	328	130	-4%

Carotenes (*μ*g)	3,768	2,084	3,724	1,765	-1%
Vitamin A (IU)	3,679	1,836	3,506	1,529	-5%
Vitamin D (IU)	430	194	421	206	-2%
Vitamin E (mg)	9.2	2.5	8.6	2.6	-6%**
Vitamin C (mg)	167	75	166	66	0%

Potassium (mg)	2,967	962	2,837	872	-4%
Calcium (mg)	705	257	676	250	-4%
Magnesium (mg)	250	68	234	69	-6%**
Phosphorous(mg)	1,120	303	1,074	320	-4%
Iron (mg)	11.2	5.0	10.3	2.9	-8%
Zinc (*μ*g)	8,796	2,223	8,492	2,301	-3%
Copper (*μ*g)	1,198	311	1,163	313	-3%

Total dietary fiber (g)	16.0	6.3	15.4	5.3	-4%
Soluble dietary fiber (g)	3.0	1.5	2.8	1.2	-5%
Insoluble dietary fiber (g)	12.2	4.8	11.7	3.9	-4%

Median					-4%

* p<0.05, ** p<0.01 by paired *t*-test.

Pearson’s CCs with log-transformation and energy adjustment ranged from 0.23 (zinc) - 0.55 - 0.74 (insoluble DF) (Table 5). ANOVA ICCs with log-transformation and energy adjustment ranged from 0.37 (zinc) - 0.70 - 0.84 (insoluble DF). Spearman’s rank CCs with energy adjustment ranged from 0.25 (zinc) - 0.56 - 0.74 (magnesium). Consistent amelioration was observed for neither Pearson’s CCs nor ICCs by log-transformation with/without energy-adjustment. Spearman’s rank CCs were not improved by energy-adjustment, either.

**Table 5.** Pearson’s CCs, ANOVA ICCs and Spearman’s rank CCs between daily intake of selected nutrients based on SQFFQ_96_ and SQFFQ_97_.

	Pearson’s CCs					ANOVA ICCs					Spearman’s rank CCs			
			
Nutrient		Log- transformed	Log- transformed, energy- adjusted	95% CIs			Log- transformed	Log- transformed, energy- adjusted	95% CIs			Energy- adjusted	95% CIs	
Energy	0.78	0.82	NA	NA	NA	0.87	0.89	NA	NA	NA	0.78	NA	NA	NA
Total protein	0.74	0.74	0.57	(0.40 - 0.57)		0.85	0.84	0.72	(0.57 - 0.82)		0.74	0.56	(0.39 - 0.56)	
Animal origin	0.68	0.68	0.52	(0.34 - 0.52)		0.81	0.81	0.68	(0.51 - 0.79)		0.69	0.36	(0.16 - 0.36)	
Vegetable origin	0.67	0.72	0.62	(0.47 - 0.62)		0.80	0.83	0.76	(0.63 - 0.84)		0.71	0.64	(0.50 - 0.64)	
Total fat	0.75	0.74	0.41	(0.21 - 0.41)		0.86	0.85	0.58	(0.35 - 0.73)		0.71	0.52	(0.35 - 0.52)	
Animal origin	0.69	0.69	0.58	(0.42 - 0.58)		0.81	0.82	0.72	(0.56 - 0.82)		0.68	0.54	(0.37 - 0.54)	
Fish origin	0.65	0.69	0.60	(0.44 - 0.60)		0.79	0.81	0.75	(0.61 - 0.84)		0.63	0.56	(0.40 - 0.56)	
Vegetable origin	0.75	0.70	0.49	(0.31 - 0.49)		0.85	0.83	0.66	(0.47 - 0.78)		0.76	0.56	(0.40 - 0.56)	
Carbohydrate	0.71	0.78	0.48	(0.30 - 0.48)		0.83	0.88	0.65	(0.45 - 0.77)		0.73	0.51	(0.34 - 0.51)	

SFAs	0.70	0.70	0.54	(0.37 - 0.54)		0.82	0.82	0.68	(0.50 - 0.79)		0.71	0.56	(0.39 - 0.56)	
MUFAs	0.74	0.72	0.44	(0.25 - 0.44)		0.85	0.84	0.61	(0.41 - 0.75)		0.70	0.51	(0.33 - 0.51)	
18:1	0.74	0.73	0.45	(0.26 - 0.45)		0.85	0.84	0.62	(0.42 - 0.76)		0.70	0.54	(0.37 - 0.54)	
PUFAs	0.73	0.71	0.41	(0.22 - 0.41)		0.84	0.83	0.58	(0.36 - 0.73)		0.73	0.52	(0.35 - 0.52)	
n-6 PUFAs	0.71	0.70	0.40	(0.20 - 0.40)		0.83	0.82	0.57	(0.33 - 0.72)		0.73	0.50	(0.32 - 0.50)	
18:2n-6	0.71	0.69	0.41	(0.21 - 0.41)		0.83	0.82	0.57	(0.34 - 0.72)		0.74	0.51	(0.33 - 0.51)	
20:4n-6	0.74	0.71	0.49	(0.31 - 0.49)		0.85	0.82	0.66	(0.48 - 0.78)		0.75	0.48	(0.30 - 0.48)	
n-3 PUFAs	0.71	0.71	0.52	(0.35 - 0.52)		0.83	0.83	0.69	(0.52 - 0.80)		0.71	0.61	(0.46 - 0.61)	
18:3n-3	0.66	0.61	0.39	(0.20 - 0.39)		0.80	0.75	0.56	(0.32 - 0.72)		0.63	0.44	(0.25 - 0.44)	
20:5n-3	0.63	0.68	0.60	(0.44 - 0.60)		0.77	0.81	0.75	(0.62 - 0.84)		0.64	0.61	(0.46 - 0.61)	
22:6n-3	0.65	0.70	0.58	(0.42 - 0.58)		0.78	0.82	0.74	(0.59 - 0.83)		0.67	0.60	(0.44 - 0.60)	
n-6 PUFAs/n-3 PUFAs	0.59	0.60	0.60	(0.44 - 0.60)		0.71	0.73	0.73	(0.58 - 0.82)		0.60	0.61	(0.45 - 0.61)	
Cholesterol	0.77	0.75	0.49	(0.31 - 0.49)		0.87	0.85	0.67	(0.49 - 0.78)		0.80	0.46	(0.27 - 0.46)	

Carotenes	0.51	0.63	0.59	(0.43 - 0.59)		0.67	0.77	0.74	(0.61 - 0.83)		0.62	0.58	(0.41 - 0.58)	
Vitamin A	0.64	0.70	0.69	(0.56 - 0.69)		0.77	0.82	0.82	(0.72 - 0.88)		0.73	0.72	(0.60 - 0.72)	
Vitamin D	0.61	0.68	0.59	(0.43 - 0.59)		0.76	0.80	0.74	(0.61 - 0.83)		0.60	0.55	(0.38 - 0.55)	
Vitamin E	0.76	0.78	0.47	(0.29 - 0.47)		0.86	0.87	0.65	(0.47 - 0.78)		0.77	0.55	(0.38 - 0.55)	
Vitamin C	0.63	0.65	0.56	(0.40 - 0.56)		0.77	0.79	0.71	(0.56 - 0.81)		0.65	0.56	(0.40 - 0.56)	

Potassium	0.71	0.72	0.66	(0.52 - 0.66)		0.83	0.84	0.79	(0.68 - 0.87)		0.74	0.67	(0.53 - 0.67)	
Calcium	0.70	0.69	0.65	(0.51 - 0.65)		0.82	0.81	0.79	(0.67 - 0.86)		0.73	0.70	(0.57 - 0.70)	
Magnesium	0.73	0.74	0.71	(0.59 - 0.71)		0.84	0.84	0.83	(0.74 - 0.89)		0.76	0.74	(0.62 - 0.74)	
Phosphorous	0.76	0.75	0.67	(0.53 - 0.67)		0.86	0.85	0.80	(0.69 - 0.87)		0.76	0.68	(0.54 - 0.68)	
Iron	0.41	0.58	0.44	(0.25 - 0.44)		0.53	0.73	0.58	(0.36 - 0.73)		0.68	0.55	(0.38 - 0.55)	
Zinc	0.56	0.62	0.23	(0.01 - 0.23)		0.71	0.76	0.37	(0.02 - 0.59)		0.58	0.25	(0.04 - 0.25)	
Copper	0.63	0.67	0.47	(0.29 - 0.47)		0.77	0.80	0.63	(0.43 - 0.76)		0.62	0.54	(0.36 - 0.54)	

Total dietary fiber	0.73	0.73	0.73	(0.61 - 0.73)		0.84	0.85	0.83	(0.74 - 0.89)		0.67	0.66	(0.52 - 0.66)	
Soluble dietary fiber	0.71	0.69	0.63	(0.49 - 0.63)		0.82	0.81	0.77	(0.64 - 0.85)		0.66	0.57	(0.40 - 0.57)	
Insoluble dietary fiber	0.73	0.75	0.74	(0.62 - 0.74)		0.84	0.86	0.84	(0.75 - 0.89)		0.69	0.69	(0.56 - 0.69)	

Median	0.71	0.70	0.55			0.83	0.82	0.70			0.71	0.56		

Agreement with energy adjustment ranged from 45% (animal protein) - 56% - 67% (vitamin A) and disagreement ranged from 2% (magnesium and insoluble DF) - 6% - 14% (animal protein) (Table 6). *Kappa* statistics with energy adjustment ranged from 0.18 (animal protein) - 0.34 - 0.46 (insoluble DF). Weighted *kappa* statistics ranged from 0.25 (zinc) - 0.50 - 0.68 (insoluble DF). The values of weighted *kappa* statistics were again universally better than with *kappa* statistics.

**Table 6.** Agreement, disagreement and *kappa* statistics according to tertile classification of daily intake of selected nutrients based on SQFFQ_96_ and SQFFQ_97_.

Nutrient	Agreement (%)	Disagreement (%)	*Kappa*	Weighted *kappa*	95% CIs	Energy-adjusted					

						Agreement (%)	Disagreement (%)	*Kappa*	Weighted *kappa*	95% CIs	
Energy	62	2	0.43	0.66	(0.54 - 0.78)	NA	NA	NA	NA	NA	NA
Total protein	65	4	0.48	0.66	(0.53 - 0.79)	57	7	0.36	0.52	(0.35 - 0.69)	
Animal origin	62	7	0.43	0.55	(0.39 - 0.72)	45	14	0.18	0.27	(0.06 - 0.48)	
Vegetable origin	61	4	0.41	0.63	(0.49 - 0.76)	61	6	0.41	0.57	(0.41 - 0.73)	
Total fat	57	5	0.36	0.57	(0.43 - 0.72)	56	8	0.34	0.48	(0.31 - 0.66)	
Animal origin	67	7	0.50	0.59	(0.42 - 0.76)	54	8	0.30	0.46	(0.29 - 0.64)	
Fish origin	52	7	0.29	0.48	(0.32 - 0.65)	54	11	0.30	0.41	(0.22 - 0.60)	
Vegetable origin	71	2	0.57	0.73	(0.62 - 0.85)	55	7	0.32	0.50	(0.33 - 0.67)	
Carbohydrate	71	5	0.57	0.68	(0.53 - 0.82)	54	6	0.30	0.52	(0.36 - 0.67)	

SFAs	67	2	0.50	0.70	(0.58 - 0.81)	57	7	0.36	0.52	(0.35 - 0.69)	
MUFAs	64	7	0.46	0.57	(0.40 - 0.74)	54	8	0.30	0.46	(0.29 - 0.64)	
18:1	58	6	0.38	0.55	(0.40 - 0.71)	51	8	0.27	0.45	(0.27 - 0.62)	
PUFAs	62	5	0.43	0.61	(0.46 - 0.75)	57	10	0.36	0.46	(0.28 - 0.65)	
n-6 PUFAs	68	4	0.52	0.68	(0.55 - 0.81)	58	8	0.38	0.50	(0.32 - 0.68)	
18:2n-6	68	4	0.52	0.68	(0.55 - 0.81)	58	8	0.38	0.50	(0.32 - 0.68)	
20:4n-6	70	4	0.55	0.70	(0.57 - 0.83)	51	8	0.27	0.45	(0.27 - 0.62)	
n-3 PUFAs	64	2	0.46	0.68	(0.56 - 0.80)	58	6	0.38	0.55	(0.40 - 0.71)	
18:3n-3	63	6	0.45	0.59	(0.43 - 0.75)	49	11	0.23	0.38	(0.18 - 0.57)	
20:5n-3	55	7	0.32	0.50	(0.33 - 0.67)	58	6	0.38	0.55	(0.40 - 0.71)	
22:6n-3	55	7	0.32	0.50	(0.33 - 0.67)	57	7	0.36	0.52	(0.35 - 0.69)	
n-6 PUFAs/n-3 PUFAs	56	4	0.34	0.59	(0.46 - 0.72)	58	4	0.38	0.61	(0.48 - 0.74)	
Cholesterol	73	1	0.59	0.77	(0.67 - 0.86)	49	11	0.23	0.38	(0.18 - 0.57)	

Carotenes	60	7	0.39	0.54	(0.37 - 0.70)	54	8	0.30	0.46	(0.29 - 0.64)	
Vitamin A	64	2	0.46	0.68	(0.56 - 0.80)	67	5	0.50	0.64	(0.50 - 0.79)	
Vitamin D	56	8	0.34	0.48	(0.31 - 0.66)	50	10	0.25	0.41	(0.23 - 0.59)	
Vitamin E	63	4	0.45	0.64	(0.51 - 0.77)	55	7	0.32	0.50	(0.33 - 0.67)	
Vitamin C	62	5	0.43	0.61	(0.46 - 0.75)	50	7	0.25	0.46	(0.30 - 0.63)	

Potassium	74	5	0.61	0.70	(0.55 - 0.84)	61	4	0.41	0.63	(0.49 - 0.76)	
Calcium	70	4	0.55	0.70	(0.57 - 0.83)	61	4	0.41	0.63	(0.49 - 0.76)	
Magnesium	63	4	0.45	0.64	(0.51 - 0.77)	62	2	0.43	0.66	(0.54 - 0.78)	
Phosphorous	63	4	0.45	0.64	(0.51 - 0.77)	64	5	0.46	0.63	(0.48 - 0.77)	
Iron	57	5	0.36	0.57	(0.43 - 0.72)	58	8	0.38	0.50	(0.32 - 0.68)	
Zinc	58	8	0.38	0.50	(0.32 - 0.68)	46	15	0.20	0.25	(0.03 - 0.47)	
Copper	49	6	0.23	0.48	(0.33 - 0.64)	56	6	0.34	0.54	(0.38 - 0.69)	

Total dietary fiber	57	5	0.36	0.57	(0.43 - 0.72)	56	4	0.34	0.59	(0.46 - 0.72)	
Soluble dietary fiber	62	5	0.43	0.61	(0.46 - 0.75)	52	7	0.29	0.48	(0.32 - 0.65)	
Insoluble dietary fiber	62	5	0.43	0.61	(0.46 - 0.75)	64	2	0.46	0.68	(0.56 - 0.80)	

Median	63	4	0.43	0.61		56	6	0.34	0.50		

## DISCUSSION

When dealing with epidemiologic data, validity and reproducibility are the two most important elements. Relative validity of an SQFFQ versus 28 day WDRs was found to be higher than the values conducted in Japan and essentially equal to those obtained in other areas of the world^5^^)^. Many articles of reproducibility of intake of foods and nutrients according to FFQs/SQFFQs have been reported using various types of questionnaire, study subjects and time frames^14^^,^^16^^)^. Here we referred reports as cited below assessing reproducibility between two questionnaires administered in one-year interval and compared their values with the present study.

With respect to consumption of foods, the values for reproducibility in this study were generally similar to or greater than those reported earlier^19^^-^^23^^)^ with some exceptions^24^^-^^29^^)^. Although we did not initially inform our study subjects about the intention to administer SQFFQ_97_, we cannot preclude the possibility that there are education/learning effects on SQFFQ_97_ due to four season consecutive 7 day WDRs. Further, figures for reproducibility were presumably improved by the fact that the participants were professional dietitians, while the wide diversity of Japanese foods may have exerted an opposing influence. Thus, Japanese FFQs/SQFFQs for foods^30^^,^^31^^)^ seem to have performed as a whole rather less well than those in other regions of the world, cited previously.

Similarly, with reference to nutrients, indices of reproducibility in the present study were generally similar to or greater than those reported around the world^19^^,^^23^^,^^29^^,^^32^^-^^40^^)^ with exceptions^25^^,^^41^^)^. By the same token, as mentioned, Japanese FFQs/SQFFQs for nutrients^42^^)^ did not seem to have performed in general well compared with those in other regions.

In this context the interval between the first and second questionnaires is important. One week or a month may be too short because the memory would be strong. In order to evade influence of memory and change of physical conditions, a three-month interval within the same season may be appropriate for assessment of repeatability. A six-month interval would introduce effects of seasonal variation in consumption of foods and nutrients^43^^-^^48^^)^. In this study we set one-year interval to delete bias owing to memory and seasonal variance; however, we cannot exclude true change due to disability/disease, which would be compatible with the trend for the longer the administration interval, the lower the value for reproducibility^14^^,^^16^^,^^49^^)^. We prepared an item inquiring whether they intentionally/unduly modified their dietary habit and lifestyle along with any changes in medical and family history within the year, but none of the study subjects answered in the affirmative.

There are several approaches for assessing reproducibility, including Pearson’s CCs, ANOVA ICCs, Spearman’s CCs, percentages of agreement/disagreement and *kappa* statistics. Each procedure has its own strengths and weaknesses. For continuous variables, Pearson’s CCs are often adopted; however, ANOVA ICCs, like de-attenuation methods in validation studies, may be appropriate because within-individual errors are taken into account. For discrete variables, agreement and disagreement analyses include errors by chance, while *kappa* statistics, further weighted *kappa* statistics, shore up such weaknesses, and consistently improve the values. For ameliorating values of CCs, log-transformation may not be particularly useful although it reduces the width of the distribution and actually transforms a skewed into a normal distribution. Further, energy adjustment does not appear to be powerful for amending the figures of CCs.

In conclusion, the reproducibility of assessed intake of foods and nutrients according to the SQFFQ from Japanese female dietitians obtained in the present study was in line with the satisfactory relative validity with an SQFFQ versus 28 day WDRs gained earlier^5^^)^. Having observed seasonal variation in consumption of foods and nutrients in four-season consecutive 7 day WDRs, we have been executing the JADE (Japanese Dietitians’ Epidemiologic) Study, making proposals to all 47 prefectural dietetic associations in Japan and securing agreement for participation from a total of 26 of these. We have now mail-administered the validated/modified SQFFQ with a request for written informed consent to members of the associations and the alumnae of several women’s colleges for nutrition in autumn 1999, 2000 and 2001. The hope is that high-fidelity data can be secured to facilitate assessment of the association between lifestyle and health/disease.
